# CircAFF4 inhibits lung cancer progression via destabilizing GPX4 and triggering ferroptosis

**DOI:** 10.1186/s13062-026-00782-8

**Published:** 2026-04-20

**Authors:** Jianing Wang, Sicheng Xin, Chuanfeng Zhang, Ning Xie, Peng Kong, Yuan Yu

**Affiliations:** 1 Department of Biochemistry and Molecular Biology, Shandong Medical and Pharmaceutical University, Yantai, Shandong 264003 P.R. China; 2 Department of Clinical Medicine, Shandong Medical and Pharmaceutical University, Yantai, Shandong 264003 P. R. China; 3 Department of Biochemistry and Molecular Biology, College of Basic Medicine, Key Laboratory of Neural and Vascular Biology of Ministry of Education, Key Laboratory of Vascular Biology of Hebei Province, Hebei Medical University, Shijiazhuang, Hebei, 050017 P.R. China; 4 Department of Chest Surgery, Yantaishan Hospital, Yantai, Shandong 264001 P.R. China

**Keywords:** CircAFF4, Lung cancer, Ferroptosis, GPX4, USP10

## Abstract

**Background:**

Lung cancer has been the most common diagnosed cancer and the leading cause of cancer-related death. Growing evidence has demonstrated that circular RNAs (circRNAs) are closely associated with the occurrence and progression of tumors. Ferroptosis is an iron-dependent form of cell death triggered by the accumulation of lipid peroxides, and it has been considered as a potential target for cancer therapy. However, the specific mechanism by which circRNAs modulate ferroptosis in lung cancer remains largely unknown.

**Methods:**

Through retrieval from The Cancer Genome Atlas (TCGA) database, the differential expression of AFF4 mRNA in lung cancer was identified. Further screening and validation revealed that circAFF4, a circular RNA derived from AFF4 host gene, also exhibited a similar trend in lung cancer. Subsequently, quantitative reverse transcription-PCR (qRT-PCR) and fluorescent in situ hybridization (FISH) were used to detect its expression pattern and distribution. Functional studies were conducted both in vitro and in vivo to determine the biological functions of circAFF4. Furthermore, the interaction between circAFF4 and ubiquitin-specific peptidase 10 (USP10) as well as the relationship between USP10 and Glutathione peroxidase 4 (GPX4), were investigated by biotin-labeled RNA pull-down, mass spectrometry, RNA immunoprecipitation (RIP), FISH and co-immunoprecipitation (Co-IP) assays.

**Results:**

Here we demonstrated that circAFF4 was significantly downregulated in lung cancer tissues and lung cancer cells. In vitro and in vivo experiments suggested that circAFF4 inhibited the proliferation of lung cancer cells and promoted ferroptosis. Mechanistically, circAFF4 bound to the deubiquitinating enzyme USP10, which in turn suppressed USP10-mediated deubiquitination of GPX4, and enhanced the ubiquitin-dependent proteasome degradation of GPX4, thereby facilitating ferroptosis in lung cancer cells.

**Conclusions:**

Our findings reveal a novel mechanism by which circAFF4 interacts with USP10, impairing USP10-mediated stabilization of GPX4, promoting ferroptosis in lung cancer cells, and ultimately suppressing lung cancer progression. The circAFF4/USP10/GPX4 axis provides a new direction and may be a potential target for lung cancer treatment.

**Supplementary information:**

The online version contains supplementary material available at 10.1186/s13062-026-00782-8.

## Introduction

Lung cancer is the leading cause of cancer-related death worldwide [1]. Lung cancer is roughly divided into small-cell and non-small-cell histologies; non-small-cell lung cancer (NSCLC) comprises > 85% of all cases and can be further classified by histological subtype [1]. According to data from the International Agency for Research on Cancer (IARC) under the World Health Organization (WHO), there were 2.481 million new lung cancer cases worldwide in 2022, accounting for 12.4% of all new cancer cases, and 1.817 million deaths from lung cancer, representing 18.7% of all cancer-related deaths [2]. Smoking is the primary risk factor for lung cancer; environmental and occupational risk factors for lung cancer, such as unprocessed biomass fuels, asbestos, arsenic, and radon, may also contribute to the incidence of lung cancer in certain regions of the world [1]. In terms of diagnosis and treatment, the widespread adoption of low-dose spiral computed tomography (CT) screening has increased the early detection rate of lung cancer to 90% [3]. With advances in medical research, lung cancer treatment has entered a new era of molecular targeted therapy and immunotherapy. The successive development of drugs targeting gene mutations such as EGFR, ALK, and ROS1 has brought hope for lung cancer treatment [4–6]. However, recurrence and disease progression due to drug resistance remain common [7]. Therefore, identifying new therapeutic targets is crucial for the treatment of lung cancer.

Ferroptosis is an iron-dependent form of cell death caused by the accumulation of lipid peroxides, which was first coined by Stockwell and his co-workers in 2012 [8–11]. Numerous recent studies have demonstrated that ferroptosis exerts a potent tumor-suppressive effect in various types of cancer. For example, targeting inhibition of ferroptosis circuit in gastric cancer cells induced by STAT3 not only suppressed the progression of gastric cancer but also alleviated chemoresistance [12]. Chemotherapeutic agents such as cytarabine (ara-C), cisplatin, doxorubicin, and temozolomide, when combined with the ferroptosis inducer erastin, exhibited a significant synergistic effect on their antitumor activity [13]. GPX4 is a key regulator of ferroptosis that utilizes glutathione (GSH) as a reducing agent to scavenge lipid peroxidation products, thereby alleviating lipid peroxidation and suppressing ferroptosis [14, 15]. Previous studies have determined that dampening the activity of GPX4 or accelerating its ubiquitination degradation through direct or indirect binding with small molecule compounds or proteins can induce ferroptosis in tumor cells [16, 17]. Therefore, GPX4 has been identified as a promising therapeutic target in ferroptosis-based cancer therapy [18].

Circular RNAs (circRNAs) are covalently closed molecules generated by the back-splicing of the 3‘and 5’ exon ends of precursor RNAs, which exhibit high exonuclease resistance, cell-type specificity, and strong cross-species conservation [19]. Growing investigations have suggested that circRNAs exert their biological functions by acting as microRNA (miRNA) sponges, RBP-binding molecules, transcriptional regulators, or protein translation templates. More importantly, accumulated evidence has demonstrated that circRNAs are involved in the occurrence and development of various tumors, especially lung cancer. However, the effects of circRNAs on ferroptosis and the definite molecular mechanisms in lung cancer has not been fully elucidated.

In this study, we identified a novel circRNA derived from exons 2, 3, 4, and 5 of the AFF4 host gene, designated as circAFF4, which is downregulated in lung cancer tissues. Additionally, through both in vitro and in vivo experiments, we determined that circAFF4 inhibited the proliferation of lung cancer cells and promoted ferroptosis. through binding to the deubiquitinase USP10, impairing USP10-mediated deubiquitination of GPX4 and stability. The disruption of circAFF4 may be a novel mechanism in the occurrence of lung cancer, and is a new biomarker and potential therapeutic target for lung cancer treatment.

## Materials and methods

### Cell culture

Lung cancer cell lines (A549, NCI-H1975, NCI-H1299) and BEAS-2B were purchased from the Shanghai Institute of Cell Biology, Chinese Academy of Sciences, China. A549, NCI-H1975 and NCI-H1299 cells were cultured in RPMI-1640 (Gibco, New York, USA) and BEAS-2B cells were maintained in DMEM medium (Gibco), supplemented with 10% fetal bovine serum (Gibco), 1% penicillin–streptomycin (Solaribio, Beijing, China). Cells were maintained at 37 °C in a 5% CO₂ atmosphere.

### RT-qPCR

RNA extraction and RT‑qPCR RNA was extracted from cells using TRIzol reagent (Takara, Tokyo, Japan), and quantified using an ultramicrovolume spectrophotometer. Reverse transcription of RNA was performed using HiScript II Q RT SuperMix for qPCR (+gDNA wiper) (Vazyme, R223). ChamQ SYBR qPCR Master Mix (Vazyme, Q311) was used for RT-­qPCR analysis. GAPDH or U6 was used as the internal control; the relative expression of RNA was calculated by 2^−ΔΔct^. RT-qPCR amplification was performed using a thermal cycler (Eppendorf, Hamburg, Germany). The primers used in this study were shown in Additional file 1: Table S1.

### Clinical specimens

A total of 23 specimens from patients with lung cancer were obtained after operation from the Yantaishan Hospital. The lung cancer and adjacent control tissues were stored at −80 °C. This study was approved by the Shandong medical and pharmaceutical university Ethics Committee. Before inclusion in the study, all patients were fully informed about the research procedure and signed informed consent forms.

### RNase R digestion and actinomycin D assay

After extracting total RNA from A549 cells, 2 μg of RNA was added to 2 U of RNase R (GENESEEO, R0301), followed by enzymatic digestion at 37 °C for 10 minutes and subsequent incubation at 70 °C for 10 minutes. Reverse transcription was then performed, and the reverse transcription products were subjected to quantitative analysis using RT-qPCR. A549 cells were cultured with actinomycin D (APEXBIO, A4448) at a concentration of 5 μg/mL, and cells were harvested at 0, 4, 8, 12, and 24 hours, respectively. After RNA extraction, RT-qPCR was used to detect RNA expression levels.

### FISH assay

The biotin-labeled oligonucleotide probe for circAFF4 (5’-Cy3-TCA TGT TGC TTA GTT GGA AAA GGAA-Cy3-3’) was commercially synthesized (GenePharme). Cell crawling slides were prepared, fixed with 4% paraformaldehyde for 15 minutes, and then hybridized with the biotin-labeled circAFF4 probe and streptavidin-Cy3 (SA-Cy3) overnight. Cell nuclei were stained with DAPI. Fluorescent images were captured under a fluorescence microscope.

### Plasmids and cell transfection

The full-length sequence of circAFF4 was amplified and subcloned into the lentiviral vector pLC5-ciR (Geneseed, Guangzhou, China) to construct the circAFF4 overexpression vector pLC5-circAFF4. The full-length human USP10 cDNA and its truncated fragments were amplified and subcloned into p3×Flag -CMV10 (Geneseed, Guangzhou, China). Cell transfection was performed using ExFect Transfection Reagent (Vazyme, T101-01) according to the manufacturer’s instructions.

### EdU incorporation assay

The EdU incorporation assay was performed using an EdU kit (Red, Elab Fluor®594; Elabscience, E-CK-A377) to evaluate cell proliferation. When cells reached 50–70% confluence in 6-well plates, EdU was added to the complete medium to a final concentration of 50 μM. The cells were incubated in an incubator for 2 hours. After incubation, the samples were fixed for 15 minutes and permeabilized for 20 minutes. Fluorescent dye was added to cover the cells, followed by incubation for 30 minutes, and then DAPI staining was performed for 5–10 minutes. After washing, EdU-positive cells (red fluorescence) were observed under a fluorescence microscope. Cell nuclei were identified using DAPI (blue fluorescence).

### CCK-8 assay

Cell proliferation was evaluated using a CCK-8 kit (Elabscience, E-CK-A362). Cells were seeded into 96-well plates at a density of 2000 cells per well. After 4–6 hours, 10 μL of CCK-8 solution was added to each well, followed by incubation for 2 hours. The absorbance of each well was measured at 450 nm, starting at 0 hours, and the absorbance measurement was repeated every 24 hours thereafter.

### Cell death manner assay

Transfected A549 cells were seeded into 96-well plates at a density of 2000 cells per 100 µL per well. After 8–12 hours, 10 µM of the apoptosis inhibitor Z-VAD-FMK, 5 µM of the necrosis inhibitor Nec-1, 10 µM of the autophagy inhibitor CQ, and 10 µM of the ferroptosis inhibitor Ferrostatin-1 were added respectively, followed by incubation for 48 hours. Subsequently, 10 μL of CCK-8 was added, and after 2 hours of incubation, the absorbance of each well was measured at 450 nm.

### ROS assay

Using the Reactive Oxygen Species Assay Kit (Solaribio, CA1410), DCFH-DA was diluted in serum-free medium at a ratio of 1:1000 to a final concentration of 10 μmol/L. The cell culture medium was removed, and an appropriate volume of diluted DCFH-DA was added. The cells were incubated in a 37 °C cell incubator for 20 minutes. The cells were washed three times with serum-free cell culture medium to thoroughly remove DCFH-DA that had not entered the cells. Then, the cells were either observed under a fluorescence microscope for image capture or transferred to flow cytometry tubes for uptake detection.

### MDA assay

Three million cells were evenly distributed in a 10 cm culture dish and subjected to different transfection treatments, respectively. After 48 hours, the medium was discarded, and the cells were washed three times with PBS. The content of MDA was detected using the Malondialdehyde (MDA) Colorimetric Assay Kit (Cell Samples) (Elabscience, E-BC-K028-M).

### GSH/GSSG assay

Cell samples transfected with different plasmids were collected, and the ratio of GSH to GSSG was detected and calculated using a GSH and GSSG detection kit (Beyotime, S0053).

### BODIPY C11 assay

A549 cells were subjected to different transfection treatments for 48 hours. The cells were then incubated with BDP 581/591 C11 working solution (L267, DOJINDO) at 37 °C for 30 minutes. After washing with HBSS, the cells were observed under a fluorescence microscope.

### Western blot

Cells were harvested and lysed in RIPA lysis buffer containing PMSF. The lysate was centrifuged at 12,000 rpm and 4 °C for 10 minutes. The protein supernatant was collected and mixed with sample buffer (Beyotime), followed by boiling for 10 minutes. The proteins were separated by electrophoresis and transferred to a membrane, which was then blocked with 5% non-fat milk for 2 hours. The membrane was incubated overnight at 4 °C with primary antibodies against GPX4 (Abmart, T56959F), USP10 (SAB, 49,700), SLC7A11 (ABclonal, A2413), SLC3A2 (ABclonal, A3658), ACSL4 (Wanleibio, WL06242), and GAPDH (ZSGB-BIO, TA-08, AB_2747414). After incubation, the membrane was incubated with corresponding secondary antibodies, including goat anti-rabbit IgG (Proteintech, sa000001-2) and goat anti-mouse IgG (Elabscience, E-AB-1001) at 4 °C for 2 hours, and visualized using ECL luminescent solution.

### RNA pull‑down and mass spectrometry

RNA pull-down assay was performed using a biotin-labeled circAFF4 probe and an RNA pull-down kit (Geneseed, P0201). Briefly, the in vitro synthesized biotin-labeled nucleic acid probe was incubated with cell lysate at 4 °C for 1 hour, followed by washing with washing buffer. The mixture was boiled with 5×loading buffer for 10 minutes and centrifuged at 3000 rpm for 1 minute to collect the supernatant. The eluted proteins were subjected to Western blot analysis or LC-MS/MS analysis. For RNA-binding proteins, after separation by sodium dodecyl sulfate-polyacrylamide gel electrophoresis (SDS-PAGE), differential protein bands were visualized via Coomassie brilliant blue staining.

### Immunofluorescence

After cell climbing slides were cultured to an appropriate density, the cells were fixed for 1 hour and permeabilized for 10 minutes. Following blocking with 1% bovine serum albumin (BSA), the cells were incubated with USP10 primary antibody overnight at 4 °C, then incubated with a fluorescently labeled secondary antibody, and cell nuclei were stained with 4’,6-diamidino-2-phenylindole (DAPI). Fluorescent images were acquired using a confocal laser scanning microscope (CLSM).

### RIP assay

RNA immunoprecipitation (RIP) assay was performed using the PureBinding® RNA Immunoprecipitation Kit (GENESEEO, P0101). Magnetic beads were incubated with 5 μg of USP10 antibody or control IgG antibody (SouthernBiotech, G1418–VC98) at 4 °C for 2 hours. Meanwhile, 1 × 10^7^ cells were collected and lysed in lysis buffer for 10 minutes. The cell lysate was mixed with the bead-antibody complex and incubated overnight at 4 °C. After incubation, RNA was eluted and extracted from the complex bound to the magnetic beads, and RT-qPCR was used to verify the enrichment of circRNA.

### Co‑immunoprecipitation (co‑IP)

Co-immunoprecipitation (Co-IP) was performed using USP10 or GPX4 specific antibodies, IgG control, and an immunoprecipitation kit (Protein A+G magnetic bead method; Beyotime, P2179S). Briefly, cells were harvested and lysed on ice for 10 minutes in IP lysis buffer supplemented with protease inhibitors, followed by centrifugation at 10,000×g for 10 minutes. The supernatant was collected and incubated with 5 μg of antibody on a rotator overnight at 4 °C. Subsequently, 20 μL of pre-washed Protein A/G magnetic beads were incubated with the lysate/antibody mixture at 37 °C for 2 hours. The beads were collected using a magnetic stand and then washed with IP lysis/wash buffer. Proteins were eluted with 100 μL of sample buffer (Beyotime), heated at 95 °C for 10 minutes, and analyzed by Western blot.

### Xenograft mouse model

Four-week-old female BALB/c nude mice and five-week-old female BALB/c-nu nude mice (NSG) were used (Hangzhou Ziyuan Laboratory Animal Technology Co., Ltd. China). The mice were randomly divided into groups, and A549 cells (1 × 10^7^ cells) were subcutaneously inoculated into the dorsal side of each nude mouse. The tumor size of each mouse was monitored and calculated using the formula: length×width^2^ ×0.5. Eight weeks later, the tumor-bearing mice were euthanized, and the transplanted tumors were excised and weighed to investigate tumor growth.

### Immunohistochemistry

Paraffin-embedded sections were dewaxed and rehydrated, then incubated overnight at 4 °C with specific primary antibodies against GPX4 (1:100, SANTA) or USP10 (1:100, SAB). Subsequently, the sections were incubated with biotin-labeled secondary antibodies at 37 °C for 1 hour. After that, the sections were stained with DAB and hematoxylin, and images were captured under a microscope.

### Statistical analysis

All data were statistically analyzed using GraphPad Prism 9. Differences between two groups and among multiple groups were tested using the t-test, two-way analysis of variance (two-way ANOVA), and multiple t-tests, respectively. *p*-value < 0.05 was considered statistically significant.

## Results

### CircAFF4 is significantly downregulated in lung cancer cells and tissues

**Fig. 1:**
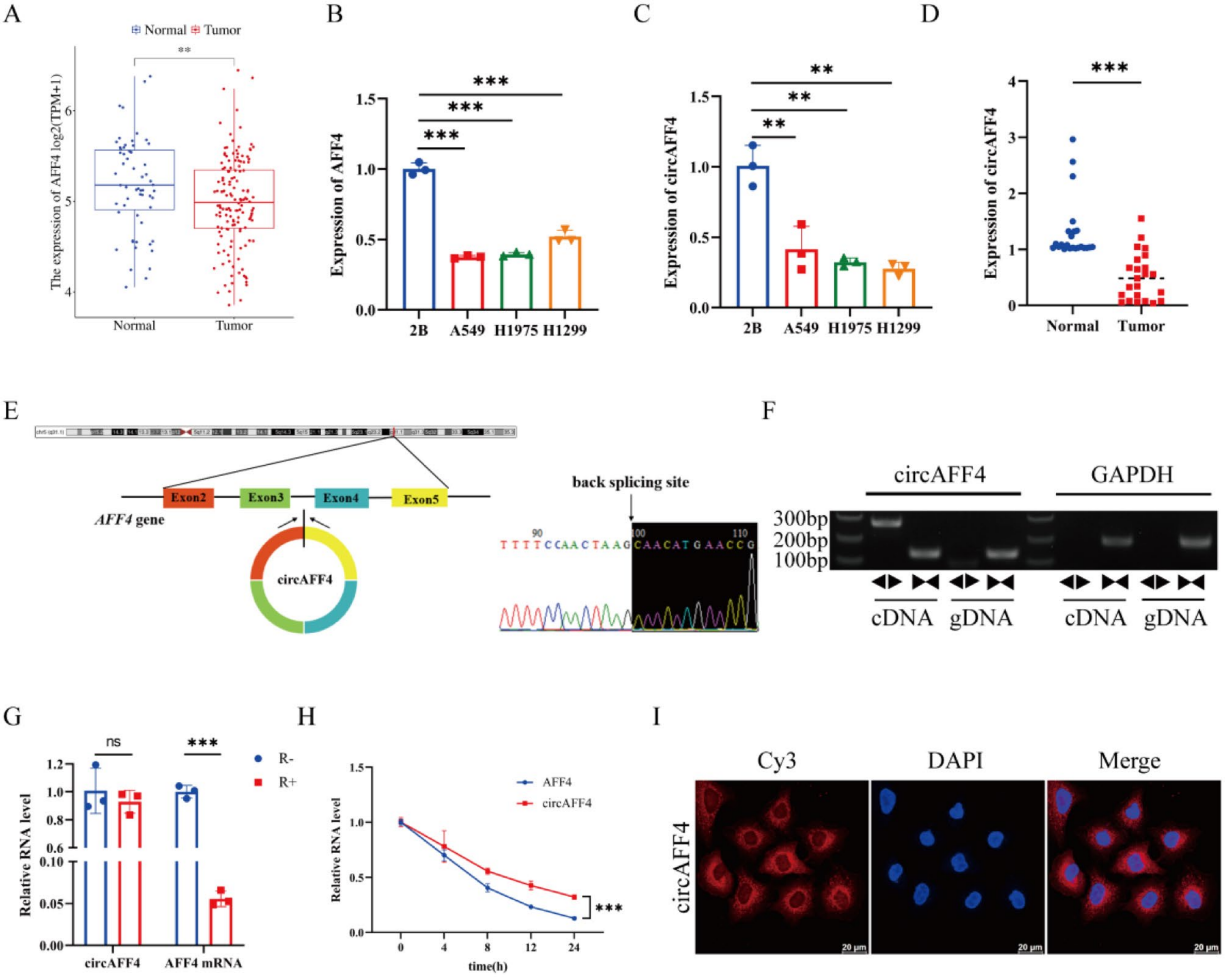
CircAFF4 is significantly downregulated in lung cancer tissues. **A** differential expression of AFF4 between lung cancer tissues (*n* = 144) and normal tissues (*n* = 59) based on bioinformatics analysis. **B** relative expression of AFF4 in lung cancer cell lines and BEAS-2B cells. **C** relative expression of circ AFF4 in lung cancer cell lines and BEAS-2B cells. **D** relative expression of circAFF4 in 23 pairs of lung cancer tissues and their adjacent normal tissues. The data were presented as the means ± SD (t-test, ***p < 0.001). **E** schematic diagram of circAFF4 formation from the AFF4 gene on chromosome 5. Sanger sequencing of the back-spliced junction of circAFF4. **F** convergent primers and divergent primers were used to amplify circAFF4 and GAPDH from gDNA and cDNA of A549 cells, respectively, followed by agarose gel electrophoresis. **G** RT-qPCR was used to detect the stability of circAFF4 and AFF4 mRNA in RNase R-treated A549 cells. **H** A549 cells were treated with act D for 0, 4, 8, 12, and 24 hours. RT-qPCR assay was performed to compare the half-lives of circAFF4 and AFF4. **I** fluorescence in situ hybridization (FISH) was used to detect the intracellular localization of circAFF4. Scale bar: 20 μm. Data are shown as mean ± SD, *n* = 3. *p < 0.05, **p < 0.01, ***p < 0.001

Through TCGA database search, we first identified the differential expression of AFF4 mRNA in lung cancer (Fig. 1A). RT-qPCR analysis determined that AFF4 mRNA expression was significantly reduced in lung cancer cell lines (A549, H1975, and H1299) compared with normal lung epithelial cell BEAS-2B (Fig. 1B). Given that previous studies have shown that circRNAs synergistically or antagonistically function with their host gene [20]. Then we examined the circAFF4 expression in lung cancer cells, and showed that circAFF4 was also remarkedly decreased in A549, H1975 and H1299 cells compared with BEAS-2B cell (Fig. 1C). Moreover, we collected 23 pairs of lung cancer tissue samples from Yantaishan hospital, and confirmed that circAFF4 had relatively low expression levels in lung cancer tissues compared with paracancerous tissues (Fig. 1D). It has been reported that AFF4 contributes into lung cancer progression [21], but the circAFF4 role in cell proliferation and death remains unclear; thus, we focused on circAFF4 in this study.

Sanger sequencing of the amplified product verified that circAFF4 is derived from exon 2 to exon 5 of AFF4 host gene (Fig. 1E). The sequence was consistent with the circBase database annotation (http://www.circbase.org/). The covalent closed-loop structure of endogenous circAFF4 was verified by RT-qPCR with convergent and divergent primers (Fig. 1F). RNase R treatment demonstrated the resistance of circAFF4 to degradation, while linear AFF4 mRNA was almost entirely degraded (Fig. 1G) which confirmed that circAFF4 harbored a closed-loop structure [22, 23]. The treatment of actinomycin D, an inhibitor of RNA synthesis, showed the stability and longer half-life of circAFF4 compared to linear AFF4 mRNA (Fig. 1H). Further investigation through RNA fluorescence in situ hybridization (FISH), confirmed that circAFF4 was mainly distributed in the cytoplasm (Fig. 1I).

### CircAFF4 inhibits the proliferation of lung cancer cells and promotes its ferroptosis

**Fig. 2:**
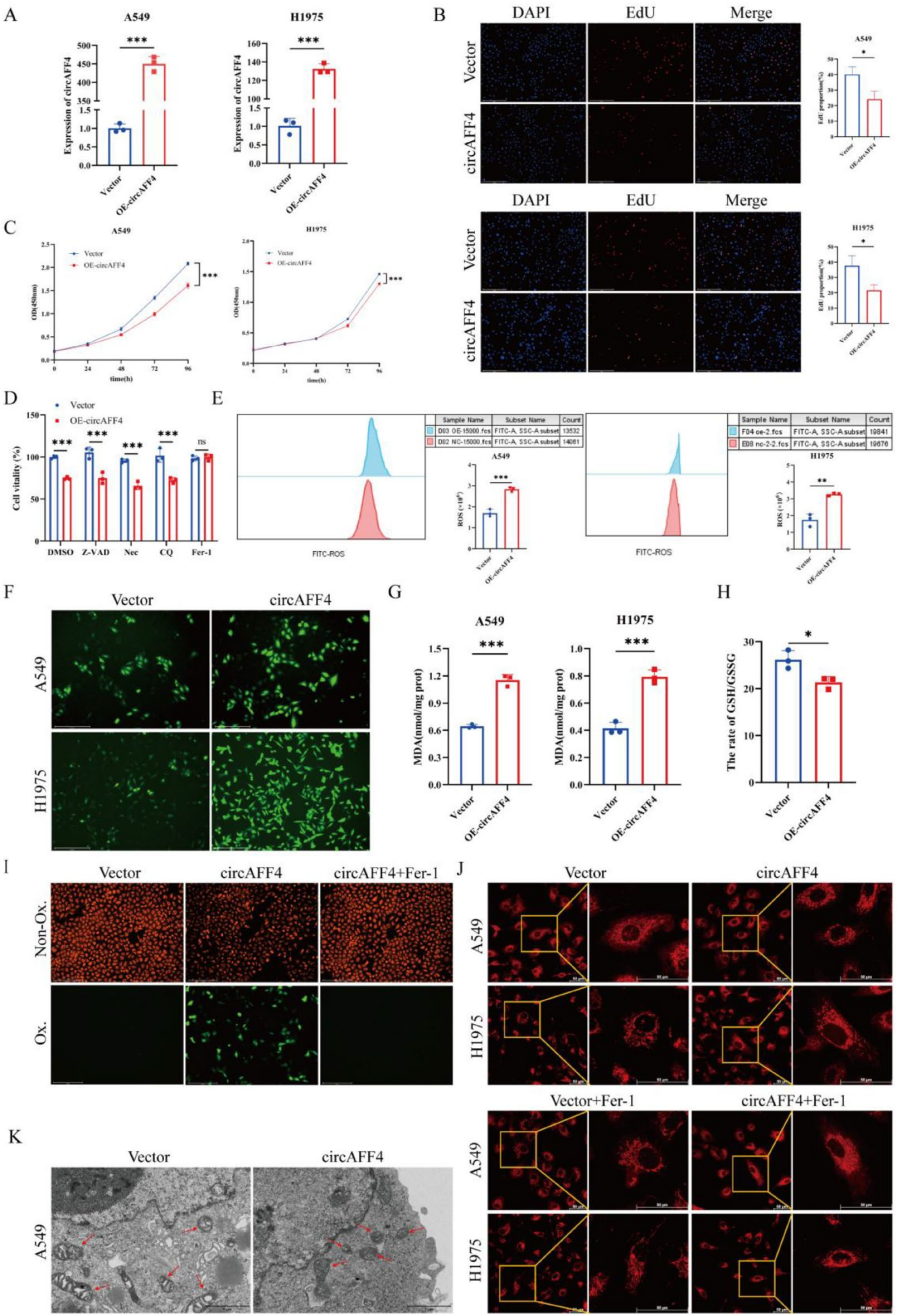
CircAFF4 inhibits the proliferation of lung cancer cells and promotes its ferroptosis. **A** qRT-PCR to determine the level of circAFF4 in A549 and H1975 cells transfected with the control, a circAFF4 overexpression vector. **B**, **C** EdU and CCK-8 assays were performed to evaluate the proliferation of A549 and H1975 cells transfected with circAFF4 or empty vector. Scale bar, 275 μm. **D** cell viability assays showed whether Z-VAD (10 μM), Nec (5 μM), CQ (10 μM) and Fer-1 (10 μM) could rescue the growth inhibition of A549 induced by circAFF4 overexpression after 48 h. **E** flow cytometry analysis of ROS production in circAFF4-overexpressing cells. **F** intracellular ROS levels in circAFF4-overexpressing cells were detected by DCFH-DA fluorescent staining. Scale bar: 275 μm. **G** intracellular MDA levels in circAFF4-overexpressing cells after 48 hours. **H** effect of circAFF4 overexpression on the GSH/GSSG ratio in A549 cells. **I** C11–BODIPY581/591 probe was used to detected lipid peroxidation level in A549 and H1975 cells transfected with circAFF4 for 48 hours by fluorescence microscope. Scale bar: 275 μm. **J** super-resolution microscopy images showing mitochondrial morphology in circAFF4-overexpressing A549 and H1975 cells after Fer-1 treatment. Scale bar: 50 μm. **K** transmission electron micrographs of circAFF4 overexpression in A549 cells for 48 h. Scale bars = 1 μm. Data are shown as mean ± SD, *n* = 3. ***p < 0.05, **p < 0.01, *p < 0.001

To investigate the biological functions of circAFF4 in lung cancer cells, we overexpressed circAFF4 in A549 and H1975 cells (Fig. 2A). EdU incorporation and CCK-8 experiments demonstrated that overexpression of circAFF4 resulted in significant suppression of cell proliferation (Fig. 2B, C). However, its corresponding linear AFF4 exhibited no such effect (Figs. A). As cell death types become increasingly diverse, tumor cell death is no longer limited to traditional necrosis and apoptosis, as novel forms such as ferroptosis and cuproptosis have been successively discovered. We further discussed the potential mechanisms by which circAFF4 inhibits the proliferation of lung cancer cells. The effects of circAFF4 on cell death by treated with Z-VAD-FMK (an apoptosis inhibitor), necrostatin-1 (a necroptosis inhibitor), CQ (an autophagy inhibitor) or Ferrostatin-1 (Fer-1, a ferroptosis inhibitor) in circAFF4-overexpressing A549 cell. The results showed that only Fer-1 treatment could reverse the inhibiting cell viability effect of circAFF4 (Fig. 2D). Treatment with the other two ferroptosis inhibitors, liproxstatin‑1 (Lip-1) and Deferoxamine (DFO), yielded similar results (Figs. B), indicating that circAFF4 may inhibit the proliferation via promoting ferroptosis.

We furtherly detected whether circAFF4 overexpression could lead to ferroptosis in lung cancer cells. Accordingly, we analyzed the levels of ROS, MDA, and the GSH/GSSG ratio, and the results showed that circAFF4 overexpression increased the levels of ROS and MDA (Fig. 2E–G), while the GSH/GSSG ratio decreased in A549 and H1975 cells (Fig. 2H). To further confirm these effects, we also detected the signal from lipid ROS. Using the C11-BODIPY probe, we found that circAFF4 overexpression increased the level of lipid peroxidation (Fig. 2I). Since the ultrastructural changes of mitochondria were considered the morphological trademark of ferroptosis [24], we observed the mitochondrial morphology of cells and showed that overexpression of circAFF4 induced mitochondrial fragmentation and shrinkage, which were reversed by the ferroptosis inhibitor Fer-1 (Fig. 2J). Furthermore, circAFF4-overexpressing cells showed that the mitochondrial membrane density was increased (Fig. 2K). Taken together, these findings indicate that the circAFF4-induced lung cancer cell viability decrease is mainly attributed to ferroptosis.

### CircAFF4 promotes ferroptosis in lung cancer cells by facilitating the ubiquitin-mediated proteasome degradation of GPX4

**Fig. 3:**
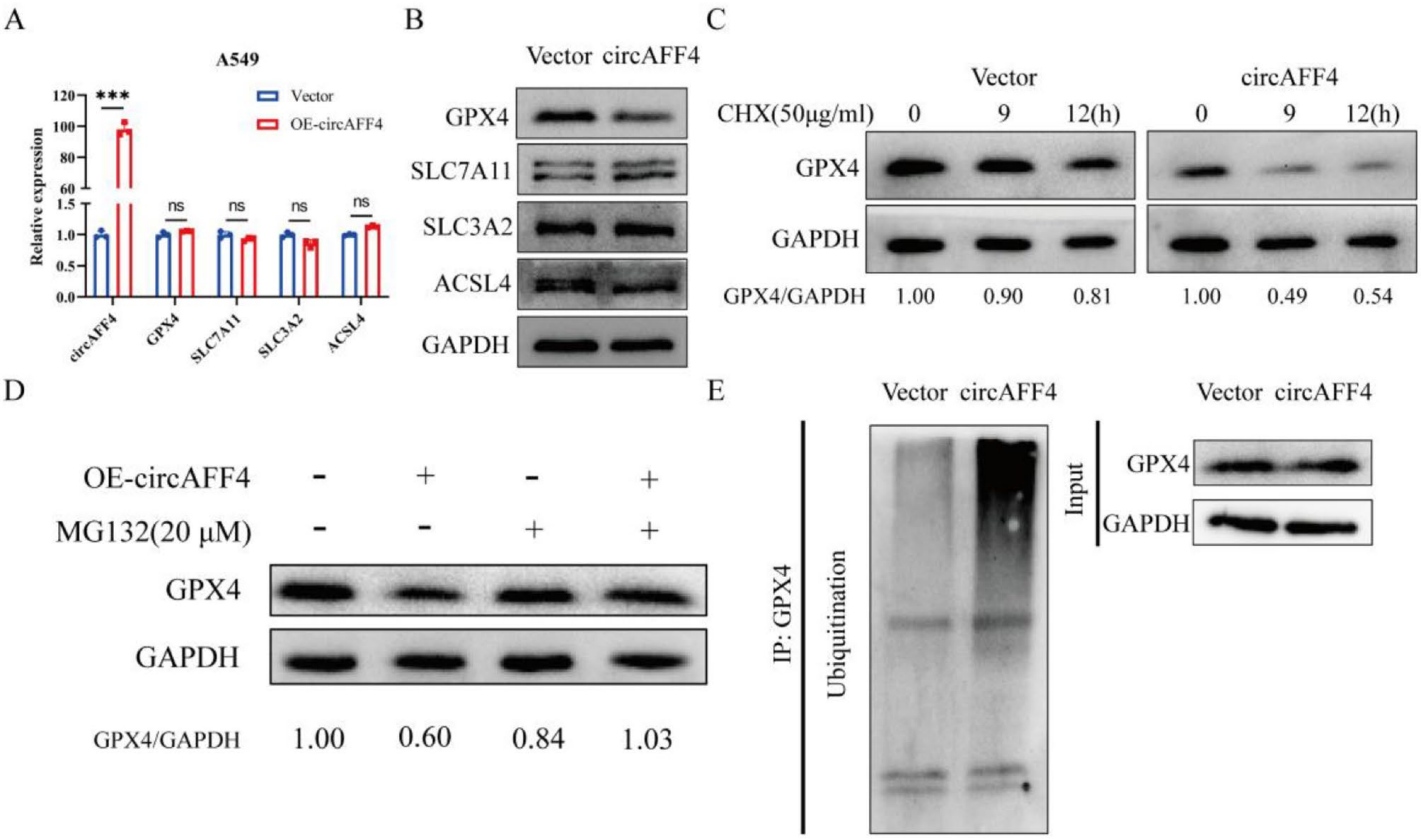
CircAFF4 promotes ferroptosis in lung cancer cells by facilitating the ubiquitin-mediated degradation of GPX4. **A** RT-qPCR was used to detect the expression of ferroptosis-related genes including GPX4, SLC7A11, SLC3A2, and ACSL4 in A549 cells 24 hours after circAFF4 overexpression. **B** Western blot was used to detect the expression levels of ferroptosis-related proteins including GPX4, SLC7A11, SLC3A2, and ACSL4 in A549 cells 48 hours after circAFF4 overexpression. **C** A549 cells transfected with circAFF4 were treated with cycloheximide (50 μM) for different time periods, and the GPX4 protein level was detected by Western blot. **D** A549 cells transfected with circAFF4 were treated with MG132 (20 μM, 8 h), and the GPX4 protein level was detected by Western blot. **E** GPX4 protein was captured by co-immunoprecipitation, and its ubiquitination level was detected by Western blot. Data are shown as mean ± SD, *n* = 3. ***p < 0.001

To explore the mechanism by which circAFF4 influences ferroptosis, we performed RT-qPCR and Western blot analysis to examine four key ferroptosis-related proteins GPX4, SLC7A11, SLC3A2, and ACSL4 at mRNA and protein levels. Interestingly, overexpression of circAFF4 did not affect their mRNA levels (Fig. 3A), but significantly decreased the protein level of GPX4 (Fig. 3B), indicating that circAFF4 induces the downregulation of GPX4 through post-translational modification. Given the protein level but not the mRNA level of GPX4 declined upon circAFF4 overexpression, we speculated that circAFF4 ma y regulate either protein stability or translation efficiency of GPX4. To test this, control or circAFF4-overexpressed cells were treated with protein translation inhibitor cycloheximide (CHX). Western blot analysis revealed that overexpression of circAFF4 reduced the stability of GPX4 protein, suggesting that circAFF4 may reduce GPX4 protein stability via enhanced proteasomal degradation (Fig. 3C). Using proteasome inhibitor, we further determined that MG-132 reversed circAFF4 induced the proteasome degradation of GPX4 (Fig. 3D). Immunoprecipitation analysis using an anti-GPX4 antibody also revealed that overexpression of circAFF4 markedly increased the ubiquitination of GPX4, further supporting that circAFF4 was involved in the stability regulation of GPX4 protein through ubiquitination (Fig. 3E). Taken together, these results indicate that circAFF4 promotes ferroptosis in lung cancer cells by facilitating the ubiquitin-mediated proteasome degradation of GPX4.

### CircAFF4 interacts with deubiquitinase USP10

**Fig. 4:**
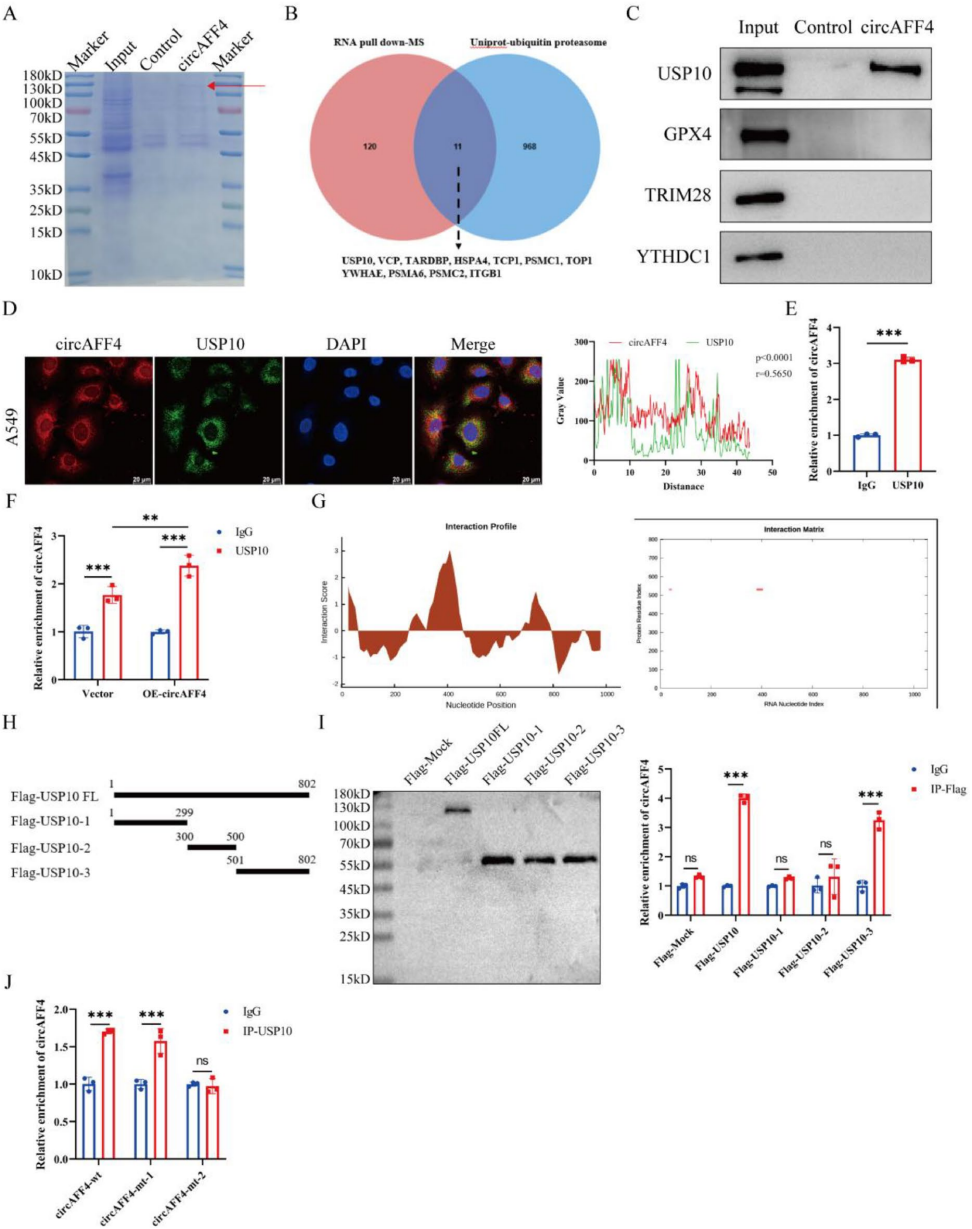
CircAFF4 directly binds to the deubiquitinase USP10. **A** Coomassie brilliant blue staining of proteins pulled down by biotin-labeled probes specific to circAFF4 and control probes. **B** venn diagram showing ubiquitin-proteasome system proteins from the uniprot database and proteins detected by mass spectrometry (MS). **C** Western blot showing that USP10 was captured by the circAFF4 probe in the RNA pull-down assay. **D** immunofluorescence-FISH was used to detect the co-localization of circAFF4 and USP10 in the cytoplasm. **E** RT-qPCR detection of RNA samples obtained from RIP assay showed that circAFF4 was captured by USP10. **F** RIP assay was performed in A549 cells using anti-USP10 antibody and IgG control under specified conditions, followed by qRT-PCR. **G** CircAFF4 binds to USP10 proteins via CatRAPID prediction. **H** schematic diagram of full-length and truncated USP10 proteins. **I** RIP assays were performed using anti-Flag antibody in A549 cells transfected with the specified full-length or truncated USP10 plasmids (containing 3×Flag tag). The co-precipitated proteins and RNA were purified, followed by Western blot and RT-qPCR, respectively. **J** RIP assay was performed in A549 cells using anti-USP10 antibody and IgG control under specified conditions, followed by qRT-PCR. Data are shown as mean ± SD, *n* = 3. ***p < 0.01, *p < 0.001

Accumulating evidence have demonstrated that circRNAs bind to specific proteins and participate in tethering certain protein-protein interactions [25]. To investigate the mechanism underlying circAFF4 promoting ferroptosis in lung cancer cells, we attempted to identify the specific protein that interact with circAFF4 in lung cancer cells. RNA pull-down assays were performed using biotin-labeled probes targeting the back-splice junction of circAFF4 in A549 cells, followed by mass spectrometry analysis (Fig. 4A). Under the cutoff criterion of unique peptides ≥ 1, we identified 131 unique proteins that were specifically pulled down by the probe targeting circAFF4. Basing on the above result that circAFF4 promoted the ubiquitin-dependent degradation of GPX4, we focus on ubiquitination-related proteins detected in the mass spectrometry screening. By cross-analyzing the mass spectrometry screening results with the ubiquitin-proteasome system proteins in Uniprot database, we selected USP10 as potential candidate protein (Fig. 4B). Next, we confirmed the interaction between circAFF4 and USP10 using RNA pull-down followed by Western blot, FISH and RIP assay (Fig. 4C–E). Additionally, overexpression of circAFF4 resulted in increased enrichment of circAFF4 by USP10 (Fig. 4F).

To explore the structural basis for the interaction between circAFF4 and USP10, the online database CatRAPID (http://s.tartaglialab.com/page/catrapid_group) was used to analyze the precise interacted structures between them. The prediction results showed that circAFF4 (26–77 nt and 376–430 nt) and the N-terminal region of USP10 (526–577 aa) displayed relatively high affinity (Fig. 4G). To verify the predicted circAFF4-binding region on USP10, we constructed Flag-tagged full-length USP10 and its deletion mutants for different functional domains (Fig. 4H). RIP assay showed that the N-terminal region of USP10 (501–802 aa), rather than other domains, was critical for the interaction with circAFF4 (Fig. 4I). Furthermore, we constructed circAFF4 deletion mutants lacking the 26-77nt (circAFF4-mt-1) and 376-430nt (circAFF4-mt-2) fragments, respectively, and performed RIP assays. The results demonstrated that the 376-430nt region of circAFF4 is critical for its interaction with USP10 (Fig. 4J). These data suggest that circAFF4 directly interacts with USP10.

### CircAFF4 inhibits USP10-mediated deubiquitination of GPX4 to promote ferroptosis in lung cancer cells

**Fig. 5:**
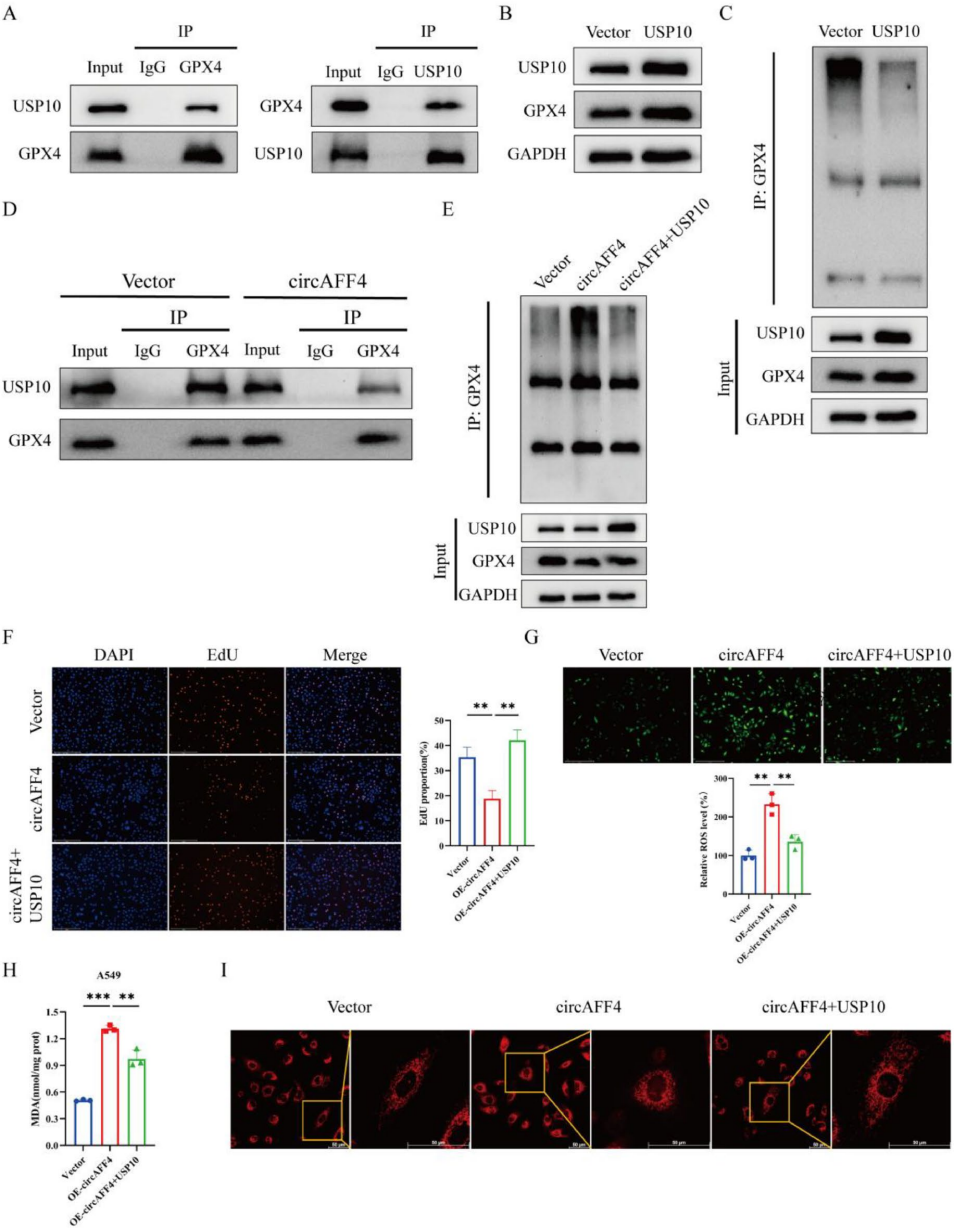
CircAFF4 inhibits USP10-mediated deubiquitination of GPX4 to promote ferroptosis in lung cancer cells. **A** Co-IP assays were performed to analyze the direct interaction between USP10 and GPX4 in A549 cells, using anti-USP10 antibody or anti-GPX4 antibody respectively. **B** Western blot assay was used to analyze the change in GPX4 protein level upon USP10 overexpression. **C** GPX4 protein was captured by co-immunoprecipitation, and its ubiquitination level was detected by Western blot. **D** Co-IP assays were performed using anti-GPX4 antibody in A549 cells transfected with the specified plasmids. **E** the ubiquitination level of GPX4 was detected in A549 cells transfected or co-transfected with the specified vectors. **F** EdU assay was used to determine the proliferative capacity of A549 cells transfected or co-transfected with the specified vectors. **G** intracellular ROS levels in cells transfected or co-transfected with the specified vectors were detected by DCFH-DA fluorescent staining. Scale bar: 275 μm. **H** intracellular MDA levels in cells transfected or co-transfected with the specified vectors after 48 hours. **I** super-resolution microscopy images showing mitochondrial morphology in cells transfected or co-transfected with the specified vectors. Scale bar: 50 μm. Data are shown as mean ± SD, *n* = 3. ***p < 0.01, *p < 0.001

USP10 has been previously reported to bind to and stabilize GPX4 in alveolar epithelial cells by direct interacting with GPX4 [26]. To determine whether this interaction exists in lung cancer cells, we performed Co-IP assays in A549 cells and found that USP10 and GPX4 co-immunoprecipitated with each other (Fig. 5A). Western blot results showed that overexpression of USP10 led to an increase in the protein level of GPX4 (Fig. 5B). Next, we examined the effect of USP10 on GPX4 ubiquitination in A549 cells, confirming that overexpression of USP10 decreased GPX4 ubiquitination (Fig. 5C). Therefore, we speculated that whether circAFF4 exerted its function by inhibiting the interaction of USP10 with GPX4. To validate this hypothesis, we further examined the potential interaction between USP10 and GPX4 with and without circAFF4 overexpression, and showed that overexpression of circAFF4 significantly reduced the abundance of USP10 precipitated by GPX4 antibody (Fig. 5D).

Ubiquitination assay also showed that the GPX4 ubiquitination was enhanced by circAFF4 overexpression, and decreased following USP10 overexpression (Fig. 5E). In addition, EdU assay showed that the proliferation-inhibitory effect induced by circAFF4 overexpression could be rescued by upregulating USP10 in A549 cells (Fig. 5F). Similarly, the increased levels of ROS and MDA, as well as mitochondrial fragmentation and shrinkage, caused by circAFF4 overexpression could be reversed by overexpression of USP10 in A549 cells (Fig. 5G–I). These results indicate that circAFF4 inhibits USP10-mediated deubiquitination of GPX4 to promote ferroptosis in lung cancer cells.

### CircAFF4 suppresses USP10-mediated deubiquitination of GPX4 to inhibit lung cancer growth in vivo

**Fig. 6:**
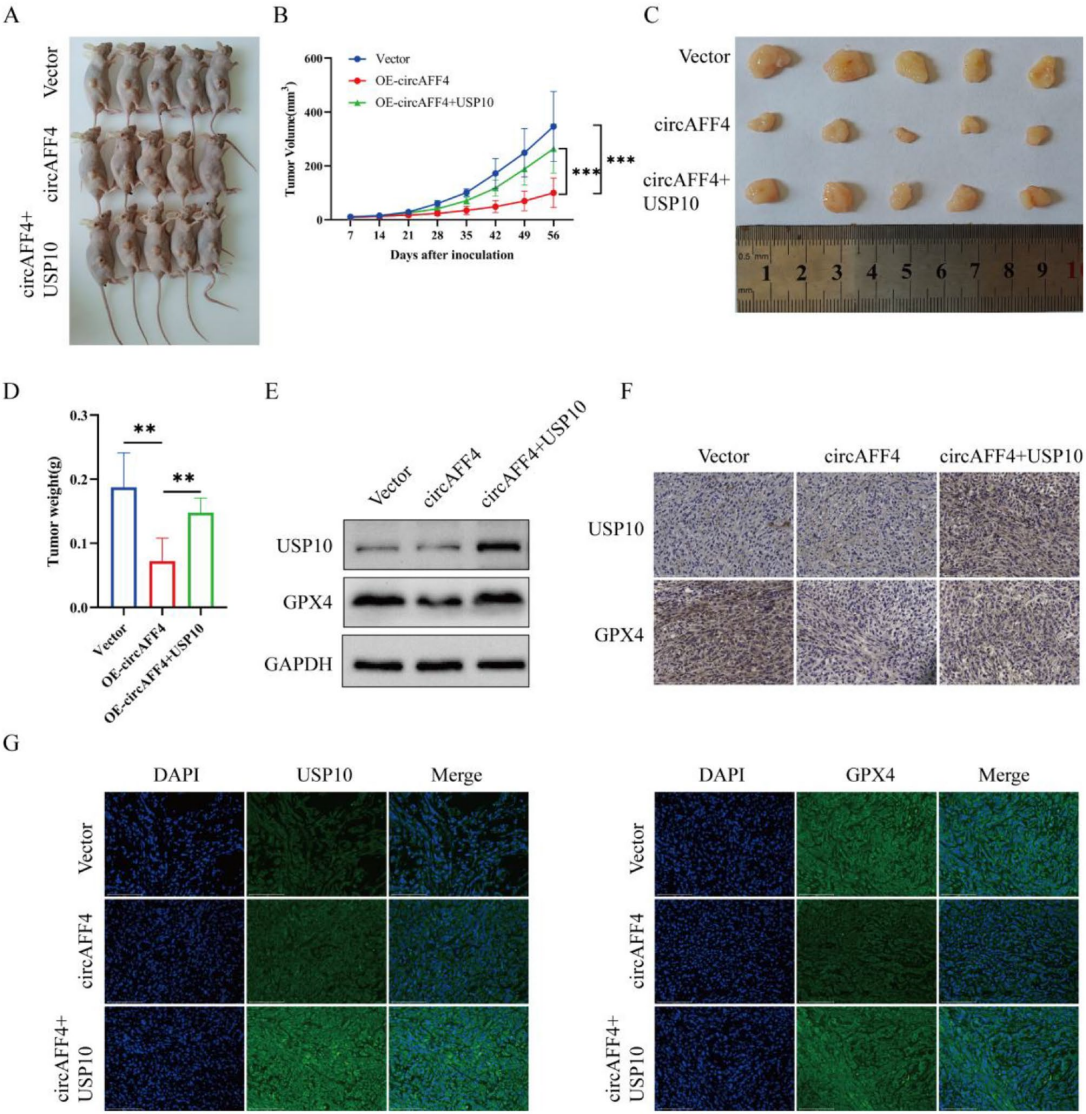
CircAFF4 inhibits USP10-mediated deubiquitination of GPX4 to suppress lung cancer growth in vivo. **A** images of tumor formation in nude mice injected with A549 cells transfected or co-transfected with specific vectors. **B** the growth curves of xenografted tumors in nude mice from each group were measured once a week. **C** images of subcutaneous tumor formation in nude mice at 8 weeks. Mice were injected with A549 cells transfected or co-transfected with specific vectors. **D** the weights of xenografted tumors in each group were calculated. **E** Western blot assay was used to detect the protein expression of GPX4 and USP10 in subcutaneous xenografts derived from A549 cells. **F**, **G** Immunohistochemistry and immunofluorescence assays were used to detect the expression of GPX4 and USP10 in xenografted tumors. Scale bar: 125 μm. Data are shown as mean ± SD, *n* = 5. ***p < 0.01, *p < 0.001

To comprehensively evaluate the effect of circAFF4 on lung cancer, we established an in vivo xenograft model in BALB/c nude mice using A549 cells with circAFF4 overexpression. Mice were randomly divided into three groups: the vector control group, the circAFF4 overexpression group, and the co-overexpression of circAFF4 and USP10 group. Tumors were induced by injecting A549 cells from different groups into the right axillary skin of mice. After 8 weeks of tumor growth tumor samples were collected (Fig. 6A). We measured the volume of subcutaneous tumors in mice and observed that overexpression of circAFF4 inhibited tumor growth, while forced expression of USP10 attenuated the tumor-suppressive effect induced by circAFF4 overexpression (Fig. 6B, C). Consistently, overexpression of circAFF4 significantly reduced tumor weight, and this effect could be reversed by overexpression of USP10 (Fig. 6D). Furthermore, Western blot, Immunohistochemistry and immunofluorescence assays showed that overexpression of circAFF4 led to a decrease in GPX4 in xenograft tumors, while overexpression of USP10 reversed this phenomenon (Fig. 6E–G). These in vivo results indicate that circAFF4 directly binds to USP10, which in turn inhibits the interaction between USP10 and GPX4, and promotes the degradation of GPX4, and thereby exerting tumor-suppressive effect in lung cancer.

## Discussion

Lung cancer ranks first globally in terms of incidence and mortality [2]. Its development is closely associated with a variety of genetic, environmental, and lifestyle factors, among which tobacco exposure is the primary risk factor for lung cancer worldwide [1]. Common treatment approaches for lung cancer include surgical resection, pharmacotherapy, and radiotherapy. Surgical resection is generally adopted for patients with early-stage lung cancer. In cases of cancer progression or metastasis, pharmacotherapy and radiotherapy usually serve as important adjuvant treatments. In recent years, the continuous advancement of immunotherapy and targeted therapy has significantly improved the efficacy of lung cancer treatment; however, recurrence and disease progression due to drug resistance remain common [7]. Therefore, identifying new therapeutic targets is of great importance. An increasing number of circRNAs have been identified to play critical roles in the occurrence and development of tumors, and have garnered significant interest due to their potential diagnostic and therapeutic value in lung cancer. Combining circRNAs with ionizable lipid nanoparticles (ILNs) can enhance immunotherapy for lung cancer [27]. Additionally, circRNAs can interact with certain proteins to regulate signaling pathways, gene transcription, and protein translation that influence the occurrence and progression of lung cancer [28–30]. The present in vitro and in vivo studies identified that circAFF4 produced from the AFF4 host gene was downregulated in lung cancer cells and tissues. Overexpression of circAFF4 was sufficient to promote ferroptosis in lung cancer cells and suppress lung cancer progression. The results shed new light on the mechanism underlying circAFF4 activity in lung cancer, which has not been previously investigated.

Ferroptosis is a novel mode of cell death characterized by increased oxidative stress caused by intracellular iron overload [31]. Inducing or inhibiting ferroptosis in lung cancer cells can significantly affect biological behaviors such as cell proliferation, migration, and invasion [32, 33]. Abnormalities in iron metabolism are often detected in lung cancer cells, leading to an overload of intracellular iron ions, which result in the generation of high ROS levels through the Fenton reaction, causing oxidative stress and cellular damage [34]. The present study demonstrated that overexpression of circAFF4 inhibits the proliferation of lung cancer cells, accompanying with increased the levels of ROS and MDA and decreased GSH/GSSG ratio, as well as the ultrastructural changes of mitochondria, all of which were considered as key trademark of ferroptosis.

In recent years, many studies indicated that GPX4 plays an essential role in the process of ferroptosis. Inhibition of GPX4 expression or activity led to ferroptosis especially in lung tumor cells [18, 35]. Being a crucial regulator of ferroptosis, GPX4 plays a role of phospholipid hydroperoxides and lowers phospholipid hydroperoxide production (AA/AdA-PE-OOH) to the phospholipid alcohol (PLOH), thereby interrupting the lipid peroxidation chain reaction. However, the mechanism of GPX4 degradation during ferroptosis still remains to be discovered. In our study, to identify potential downstream mediators of circAFF4-induced ferroptosis in lung cancer cells, we first observed that circAFF4 regulated the protein level of GPX4 without affecting its mRNA level. Furthermore, we demonstrated that circAFF4 promoted the degradation of GPX4 via the ubiquitin-proteasome pathway. Subsequently, we investigated how circAFF4 regulates the ubiquitination of GPX4, and the deubiquitinase USP10 drew our attention. After experimental verification, we discovered that circAFF4 appeared to affect the stability of GPX4 protein by interacting with USP10, leading to the degradation of GPX4 via the ubiquitin-proteasome pathway. Interestingly, the interaction between USP10 and GPX4 was attenuated following circAFF4 overexpression. Rescue experiments performed both in vitro and in vivo further verified that circAFF4 directly interacted with USP10 to inhibit USP10-mediated deubiquitination of GPX4. This inhibition subsequently promotes the ubiquitin-mediated degradation of GPX4, thereby inducing ferroptosis in lung cancer cells and ultimately suppressing lung cancer progression.

Although significant progress has been made in this study, there are several considerations that need attention. Importantly, the upstream regulatory mechanism of circAFF4 remains unclear. Whether circAFF4 regulates the proliferation of lung cancer cells through other mechanisms remains to be further investigated. Additionally, the binding site of circAFF4 that interacts with the USP10 protein is unknown and requires further investigation. To gain a more thorough and accurate understanding, further research is necessary. In summary, our data demonstrate that overexpression of circAFF4 inhibits the proliferation of lung cancer cells while promoting ferroptosis in lung cancer cells by regulating the ubiquitination level of GPX4. These findings provide a new perspective for future research on lung cancer treatment.

## Electronic supplementary material

Below is the link to the electronic supplementary material.

Supplementary Material 1

Supplementary Material 2

Supplementary Material 3

Supplementary Material 1

Supplementary Material 5

Supplementary Material 6

## Data Availability

No datasets were generated or analysed during the current study.
