# Supplementary material for: CircAFF4 inhibits lung cancer progression via destabilizing GPX4 and triggering ferroptosis
Source: Biol Direct. 2026 Apr 20;21:83. doi: 10.1186/s13062-026-00782-8 (PMC13224429; doi:10.1186/s13062-026-00782-8)
Supplement: Supplementary file 1 — Supplementary Material 1 [file 13062_2026_782_MOESM1_ESM.docx]

**Table S1. Sequences of primers used in this study.**

| Gene | Primer sequences |
| --- | --- |
| circAFF4 (Divergent) | F: 5’-TCTGAGCACTACAGCAGCCAA-3’ |
|  | R: 5’-GTTCTTTCATACGCAGCACATTC-3’ |
| circAFF4 (Convergent) | F: 5’-AAAGGCCAGCATGGATCAGAA-3’ |
|  | R: 5’-GTGATTTGGAGCGTTGATGTTC-3’ |
| AFF4 | F: 5’-AAAGGCCAGCATGGATCAGAA-3’ |
|  | R: 5’-GTGATTTGGAGCGTTGATGTTC-3’ |
| GAPDH | F: 5’-GTGAACCATGAGAAGTATG-3’ |
|  | R: 5’-CGGCCATCACGCCACAGTTTC-3’ |
| GPX4 | F: 5’-GAGGCAAGACCGAAGTAAACTAC-3’ |
|  | R: 5’-CCGAACTGGTTACACGGGAA-3’ |
| SLC7A11 | F: 5’-TCATTGGAGCAGGAATCTTCA-3’ |
|  | R: 5’-TTCAGCATAAGACAAAGCTCCA-3’ |
| SLC3A2 | F: 5’-TACCCTCTAACCCTGTTC-3’ |
|  | R: 5’-CCCGTTTCTACTGTAACC-3’ |
| ACSL4 | F: 5’-TCCAAGTAGACCAACGCCTT-3’ |
|  | R: 5’-TATGTGTCCTTCGGTCCCAG-3’ |
