# Supplementary material for: CircAFF4 inhibits lung cancer progression via destabilizing GPX4 and triggering ferroptosis
Source: Biol Direct. 2026 Apr 20;21:83. doi: 10.1186/s13062-026-00782-8 (PMC13224429; doi:10.1186/s13062-026-00782-8)
Supplement: Supplementary file 2 — Supplementary Material 2 [file 13062_2026_782_MOESM2_ESM.docx]

Supplemental data For

**CircAFF4 inhibits lung cancer progression via destabilizing GPX4 and triggering ferroptosis**

Jianing Wang1, Sicheng Xin2, Chuanfeng Zhang1, Ning Xie4, Peng Kong3*, Yuan Yu1*

1Department of Biochemistry and Molecular Biology, Binzhou Medical University, Yantai 264003, Shandong, P.R. China, 2Department of Clinical Medicine, Binzhou Medical University, Yantai 264003, Shandong, P. R. China, 3Department of Biochemistry and Molecular Biology, College of Basic Medicine, Key Laboratory of Neural and Vascular Biology of Ministry of Education, Key Laboratory of Vascular Biology of Hebei Province, Hebei Medical University, Shijiazhuang 050017, Hebei, P.R. China, 4Department of Chest Surgery, Yantaishan Hospital, Yantai 264001, Shandong, P.R. China

*Correspondence address. Tel: +86-15130176117; E-mail: pengkong@hebmu.edu.cn / Tel: +86-18660073150; E-mail: yuyuan@bzmc.edu.cn

**Thi****s file includes:**

Supplemental Figure

**Supplemental Figure**


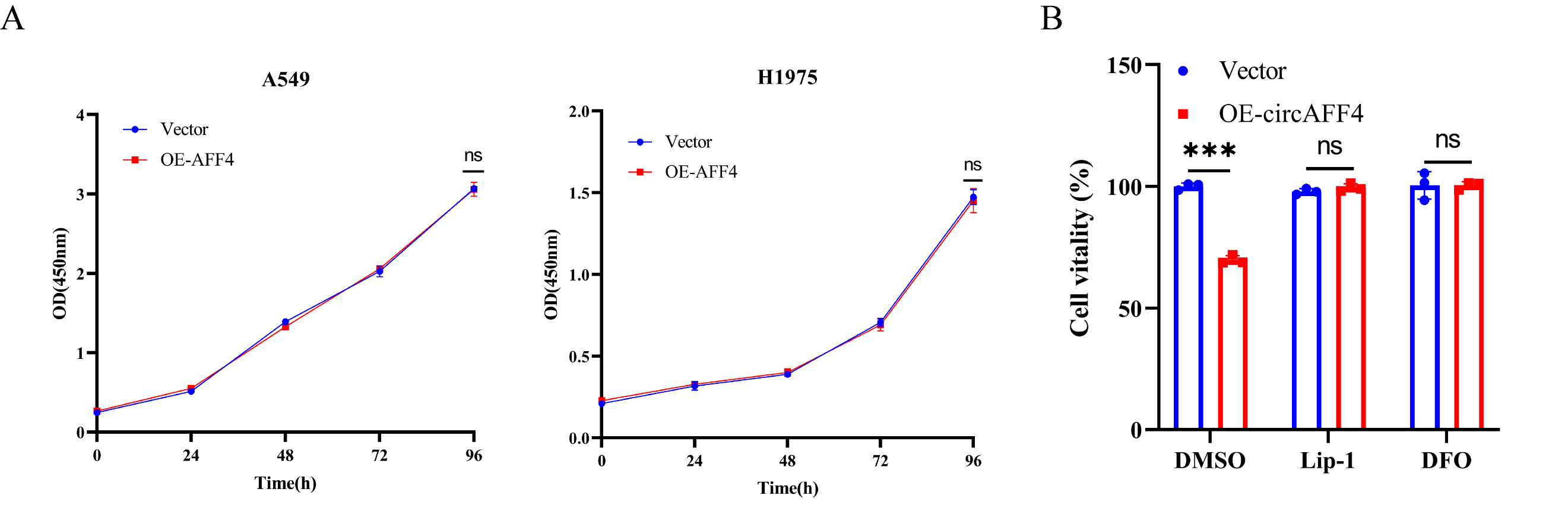


**Supplemental Figure** CircAFF4 inhibits the proliferation of lung cancer cells and promotes its ferroptosis (related to Figure2). **A** CCK-8 assay was performed to evaluate the proliferation of A549 and H1975 cells transfected with AFF4 or empty vector. **B** Cell viability assays showed whether Lip-1 (5 μM), DFO (100 μM) could rescue the growth inhibition of A549 induced by circAFF4 overexpression after 48h. Data are shown as mean ± SD, n = 3. ***P < 0.001.
