# Supplementary figures and images for: CircAFF4 inhibits lung cancer progression via destabilizing GPX4 and triggering ferroptosis
Source: Biol Direct. 2026 Apr 20;21:83. doi: 10.1186/s13062-026-00782-8 (PMC13224429; doi:10.1186/s13062-026-00782-8)

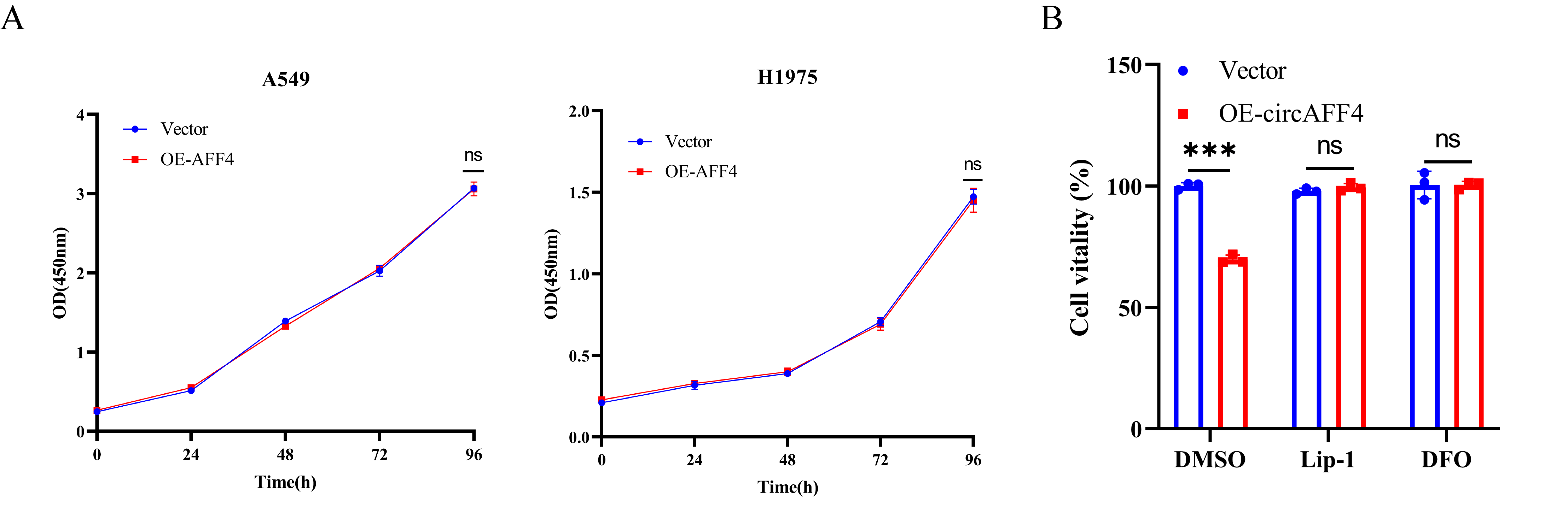

Supplement: Supplementary file 3 — Supplementary Material 3 [file 13062_2026_782_MOESM3_ESM.tif]
